# Supplementary material for: Powering AI at the edge: A robust, memristor-based binarized neural network with near-memory computing and miniaturized solar cell
Source: Nat Commun. 2024 Jan 25;15:741. doi: 10.1038/s41467-024-44766-6 (PMC10811339; doi:10.1038/s41467-024-44766-6)
Supplement: Supplementary file 1 — Supplementary Information [file 41467_2024_44766_MOESM1_ESM.pdf]

Supplementary information: Powering AI at the Edge: A Robust, Memristor-based Binarized Neural Network with Near-Memory Computing and Miniaturized Solar Cell

Fadi Jebali[1], Atreya Majumdar[2], Clément Turck[2], Kamel-Eddine Harabi[2], Mathieu-Coumba Faye[1,4], Eloi Muhr[1], Jean-Pierre Walder[1], Oleksandr Bilousov[3], Amadéo Michaud[3], Elisa Vianello[4], Tifenn Hirtzlin[4], François Andrieu[4], Marc Bocquet[1], Stéphane Collin[2,3], Damien Querlioz[2,*], and Jean-Michel Portal[1,*]

[1]Aix-Marseille Université, CNRS, Institut Matériaux Microélectronique Nanosciences de Provence, Marseille, France.
[2]Université Paris-Saclay, CNRS, Centre de Nanosciences et de Nanotechnologies, Palaiseau, France.
[3]Institut Photovoltaïque d'Ile-de-France (IPVF), Palaiseau, France.
[4]Université Grenoble Alpes, CEA, LETI, Grenoble, France.
[*]damien.querlioz@c2n.upsaclay.fr, jean-michel.portal@univ-amu.fr

Contents

Supplementary note 1: Power management unit and digital control circuitry of the binarized neural network 1
Supplementary note 2: Memristor forming and programming methodology of the binarized neural network 3
Supplementary note 3: Pipelined inference operation of the binarized neural network 4
Supplementary note 4: Resource-efficient binarized neural network mapping strategy 6
Supplementary note 5: Comparison with state-of-the-art analog in-memory computing approaches 7
Supplementary note 6: Analysis of the error sources 10
Supplementary note 7: Estimation of the error rate of an in-memory computing solution 12
Supplementary note 8: Harvester as a stable power source 13
Supplementary References 14

Supplementary note 1: Power management unit and digital control circuitry of the binarized neural network

This note describes the power management unit and the digital control circuits (finite state machines) integrated on-chip in our binarized neural network integrated circuit. These circuits are presented in Suppl. Fig. 1a.

The unique power management unit, illustrated in Suppl. Fig. 1d, routes the three power supply pads of our design (with voltages VH = 4.5 volts, VM = 2.7 volts, and VDD) to the supply voltages VDDC and VDDR, which are distributed to the memristor arrays of our system, depending on digital selection signals coming from the state machine (V_{CTRL}) . The power management unit is biased at five volts and is composed of two decoder-like circuits: one for VDDR and one for VDDC. These

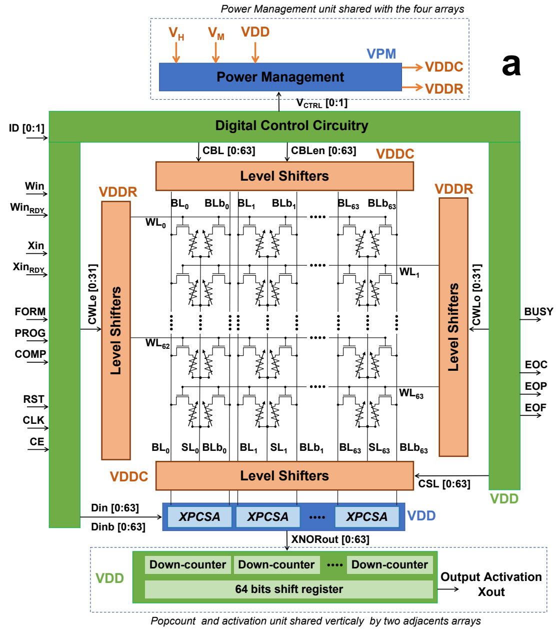

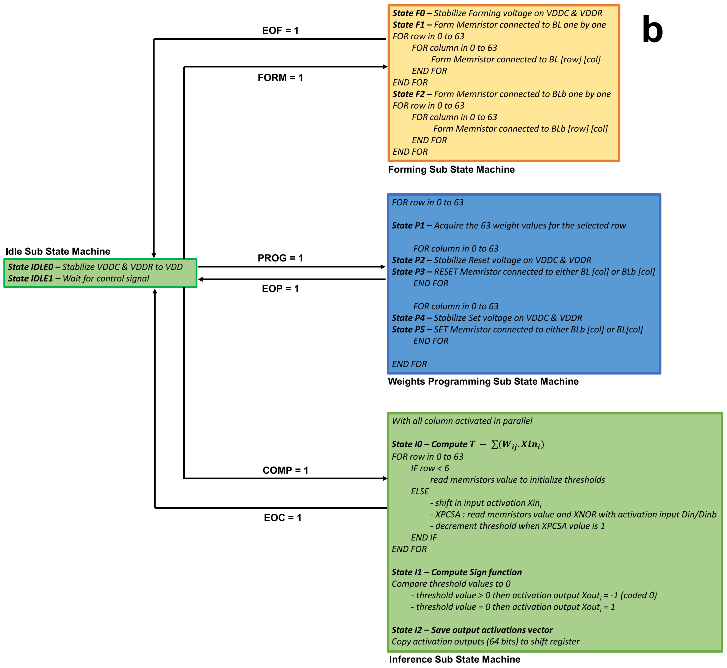

c

Pin Name	Type	Description
ID [0:1]	Input	Select the array to perform a forming or programming operation
Win	Input	Serial input for weight values
Win_{RDY}	Input	Acknowledgement to be activated when the 64 weight bits have been downloaded
Xin	Input	One bit activation input
Xin_{RDY}	Input	Acknowledgement to be activated when the activation bit has been downloaded
FORM	Input	To be activated to start the forming process on the full-array
PROG	Input	To be activated to start the weight programing process on the full-array
COMP	Input	To be activated to initiate the inference process on the full array
RST	Input	Reset the full chip
CLK	Input	Clock to synchronize the digital part of the Binary neural network
CE	Input	Enable the Binary neural network
$V_{CTRL[0:1]}$	Output	Selection bits for the power management unit
Din [0:63]	Output	Extension of the Xin value to 64 bits
Dinb [0:63]	Output	Extension of the not(Xin) value to 64 bits
CWLe [0:31]	Output	Level shifters inputs to control WL voltage of even row
CWLo [0:31]	Output	Level shifters inputs to control WL voltage of odd row
CBL [0:63]	Output	Level shifters inputs to control BL voltage
CBLen [0:63]	Output	Level shifters inputs to control BL connection to BL level shifters
CSL [0:63]	Output	Level shifters inputs to control SL voltage
BUSY	Output	Remains at 1 when sub state machine activated
EOC	Output	End of inference operation
EOF	Output	End of Forming operation
EOP	Output	End of Programming operation

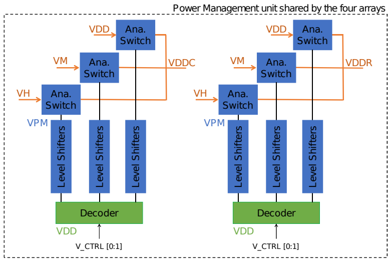

	Forming	Set	Reset	Inference
VDDC	VH	VM	VM	VDD
VDDR	VM	VM	VH	VDD

Supplementary Figure 1. Overview of the digital control circuitry associated with each memristors array a Scheme of the digital Moore state machine, including inputs and outputs pins, and control signal provided to the memristors array. The power management unit is shared by the four arrays, and the digital popcount and activation unit is shared vertically by two arrays **b** Presentation of the state machine through the three sub state machine associated with each modes of operation: forming, weight programming and inference. **c** Overview and short description of the pins of the state machine. **d** VDDC and VDDR voltages setting accordingly to the three modes of operations. The VDDC and VDDR setting is performed using the power management unit. The power management unit is composed, for both VDDC and VDDR, of a decoder-like circuit that activates one of the level shifters, among the three available, to drive the selected analog switch to connect the power supply output (VDDC/VDDR) to the selected power supply input (VH, VL, VDD).

circuits control level shifters that drive analog transmission gates (nMOS and pMOS) to connect the appropriate input power supply to VDDC and VDDR. By default, VDDC and VDDR are both connected to VDD.

The on-chip digital control circuit is divided into four similar Moore state machines, each associated with a memristor array, and placed and routed around it. This digital part of the chip is constructed using 13,842 digital gates. The state machine scheme for one memristor array is illustrated in Suppl. Fig. 1a. The state machine uses four sub-state machines to perform the four main operations on the array: forming, weight programming, inference, and idle, as presented in Suppl. Fig. 1b. The digital finite state machine circuitry directly controls the memristor arrays by providing digital signals to the level shifters that control all the lines (BL, BLb, WL, and SL) of the array. All operations, regardless of the mode, are performed row-by-row sequentially (no random access is provided) and are finished when the entire array has been scanned. Any change to the memristors during forming or programming is performed column-by-column for a given row and a given array. Conversely, all columns and all arrays are operated in parallel during the inference process for a given row.

The forming sub-state machine begins by activating the signal FORM on the selected array (signal ID). It comprises three

states: F0, a wait state that allows for stabilized VDDC and VDDR voltages in forming mode (Suppl. Fig. 1d); F1 and F2, which are dedicated to performing the form operation on the memristors connected to BL and BLb, respectively (see also Suppl. Note 2). The forming operation is executed row-by-row and column-by-column, and only once in the die's lifetime. Once all the memristors in the array have been formed, the signal EOF is set, and the machine returns to the idle state.

The programming sub-state machine is composed of five states. In state P1, for each row in the array, the weight bit stream of sixty-four bits to be programmed is shifted in serially through the input pin Win, and the programming operation of the active row begins with the acknowledgment of this shift operation with the signal $\mathrm{Win}_{\mathrm{RDY}}$. In state P2, the VDDC and VDDR voltages are set with the power management unit to their Reset values (Suppl. Fig. 1d). In state P3, column-by-column, the memristors are reset according to the weight bits, either to the memristor connected to BL or the one connected to BLb (see also Suppl. Note 2). The states P4 and P5 are similar to P2 and P3, respectively, but perform a set operation on either the memristor connected to BLb or the one connected to BL (see also Suppl. Note 2 and Suppl. Fig. 1d for VDDC and VDDR voltage values). Once the full array has been programmed with the weight values, the signal EOP is activated, and the machine returns to the idle state. The programming operation is typically performed only once per application.

Finally, the digital control circuit includes two main sub-state machines: the idle machine and the inference machine. Both share the same voltage settings (by default, VDDC and VDDR are equal to VDD), and in this case, all the arrays are operated in parallel (signal ID is disconnected). The inference process begins with the activation of the control input COMP. In this mode, all columns of the four arrays are operated in parallel. First, in state I0, the array is read row-by-row. The first six rows are used to set the initial threshold value of the neuron registers (Down-counters). For the remaining rows, the XNOR operation is performed between the input activation (vectors Din and Dinb are extensions of the Xin input on sixty-four bits) and the weight, and the threshold value is updated accordingly. The row changes when the acknowledgment signal $\mathrm{Xin}_{\mathrm{RDY}}$ is activated with the acquisition of a new activation input bit on the pin Xin. Once all 64 rows have been selected sequentially, in state I1, the 64 output activations are computed and saved during state I2 in an output shift register. The signal EOC is then asserted, and the machine returns to the idle state.

Supplementary note 2: Memristor forming and programming methodology of the binarized neural network

This note describes the method for programming memristors in the fabricated binarized neural network integrated circuit. Our system stores weights using a two-transistors/two-memristors (2T2R) structure (illustrated in Suppl. Fig. 2a), where the bit cell is composed of two memristors and their associated selection nMOSFETs. The two memristors have their bottom electrode connected to the bit cell terminals BL and BLb, respectively, through the nMOSFET selection transistors. The gates of each nMOSFET selection transistor are connected to the WL line, which allows selecting the active row in the array and limiting the current flowing through the selected memristor when necessary (e.g., during forming and SET operations). The top electrode of the memristors is connected to the SL terminal. The BL, BLb, and SL terminals are connected to vertical lines in the array, whereas the WL terminal is connected to a horizontal line in the array.

All lines in the array (BL, BLb, WL, SL) are controlled by dedicated level shifters (illustrated in Suppl. Fig. 2b). The horizontal line level shifters are powered between VDDR and ground (GND), whereas the vertical ones are powered between VDDC and ground (GND). This topology implies that during a forming or a programming operation, the vertical lines (BL, BLb, and SL) can be biased either at VDDC or ground, and the horizontal line (WL) can be biased either at VDDR or ground. These biasing conditions are defined by the control signals coming from the digital state machine (CBL, CSL, CWL). Depending on the operation to be performed, the finite state machine also defines the power supply (VH, VM, VDD) connected to VDDC and VDDR (see Suppl. Note 1), since memristors are programmed with voltages higher than the nominal voltage used for digital circuitry. When the chip is in idle state, all the lines in the array are grounded.

Suppl. Fig. 2c illustrates the voltage levels in the array for a given cell. As previously mentioned in Suppl. Note 1, during forming or programming states, the operations are performed memristor-by-memristor on the active row. It is worth noting that the voltages on BL and BLb are always complementary during a forming or programming operation (VDDC/GND or GND/VDDC). For a forming operation, a high voltage (VH) is applied to the top electrode (TE) of the memristor, and thus to the SL (VDDC=VH), while the bottom electrode (BE) is grounded. Since BL and BLb are complementary, the other memristor in the selected bit cell remains unchanged as both of its electrodes have the same potential VH. Additionally, the current flowing through the selected memristor must be carefully controlled to avoid damaging the device, thus the WL voltage is set to VDDR=VM.

The SET operation is similar to the forming operation, with a reduced voltage applied on the top electrode (TE), resulting in the SL voltage being set to VDDC=VM. The bottom electrode (BE) of the selected memristor is grounded, and the unselected

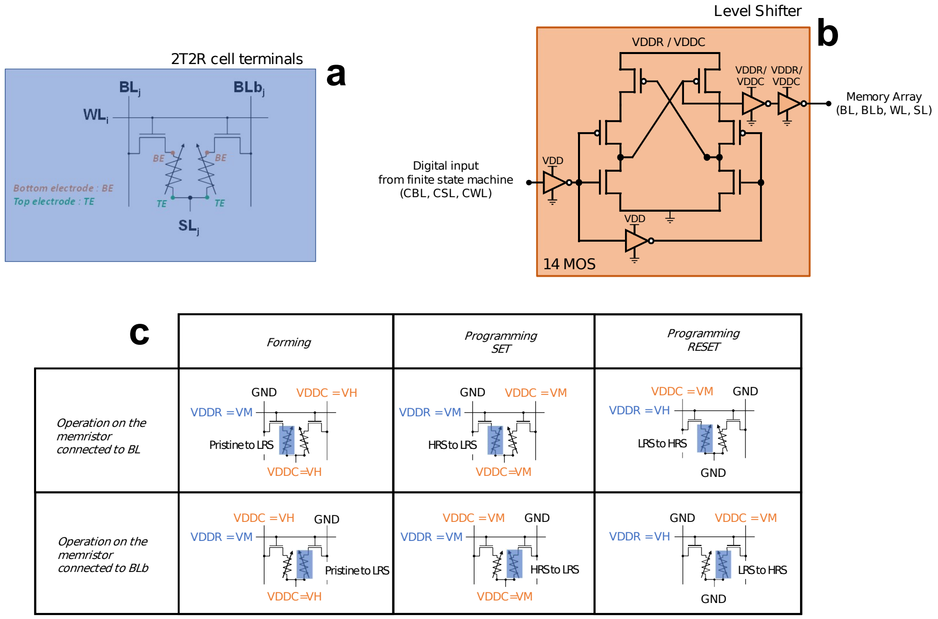

Supplementary Figure 2. Memristor forming and programming methodology of the binarized neural network. a Schematics of the 2T2R bit cell, with a focus on the bit cell architecture and the access terminals (BL, BLb, SL, WL) identification. **b** Detailed schematics of the level shifter circuit used to apply proper voltage values on the different array line (with BL, BLb, SL driven by VDDC powered level shifter and WL by VDDR powered level shifter) controlled by the digital input value provided by the digital finite state machine. **c** Voltages applied on the selected bit cell depending on the operation to be performed. Forming or programming operations are performed a single memristor at the time.

memristor has a bottom electrode biased to VM, resulting in both electrodes at the same potential. As in the forming operation, the current flowing through the device during the SET operation is limited by applying VDDR=VM on the selected WL.

In contrast, for a RESET operation, the bottom electrode (BE) of the selected memristor is set to VDDC=VM, and its top electrode is grounded through SL, meaning that the unselected memristor has both of its electrodes grounded and remains unchanged. To properly RESET a memristor, the current flowing through the device must be larger than the compliance current used to SET the device. Thus, the WL voltage is set to VDDR=VH to completely open the selection nMOSFET. Finally, the two memristors are programmed in complementary states, either LRS/HRS or HRS/LRS, to store the binary value of the weight.

Supplementary note 3: Pipelined inference operation of the binarized neural network

This note details the inference process at the circuit level, beginning with the description of the XNOR operation and followed by the population count (popcount) and threshold operations. The chip is powered at VDD volts in both idle and inference states, meaning that VDDR=VDDC=VDD. The inference process for all neurons in the arrays is performed in parallel through the successive selection of each WL in the array. As depicted in Supplementary Note 1, a single input activation bit (Xin) is received by the subsystem (two vertical arrays) and extended to 64 bits (all bits are equal to Xin) to be applied in parallel to the XNOR layer of each XPCSA. The extended vectors generated from Xin are Din and Dinb, as the XNOR layer requires both values to operate (see Supplementary Figure 3b). The read operation, in conjunction with the activation of the XNOR layer, allows for the computation of the XNOR between the input activation and the weights.

To perform the inference process, the following steps are fulfilled:

- The selected WL is activated by applying VDDR=VDD on the gate of the selection nMOSFET.
- The state machine starts the charge phase of the BL and BLb through the PCSA by setting the SEN signal to ground (CSL=1), while the SL line is also charged through the level shifter that drives SL (see Supplementary Figure 3a). This results in both BL and BLb on one side, and SL on the other side, seeing their voltages ramping up to VDD. This precharge ensures that the selected memristors in the 2T2R cell (see Supplementary Figure 3b) have a potential

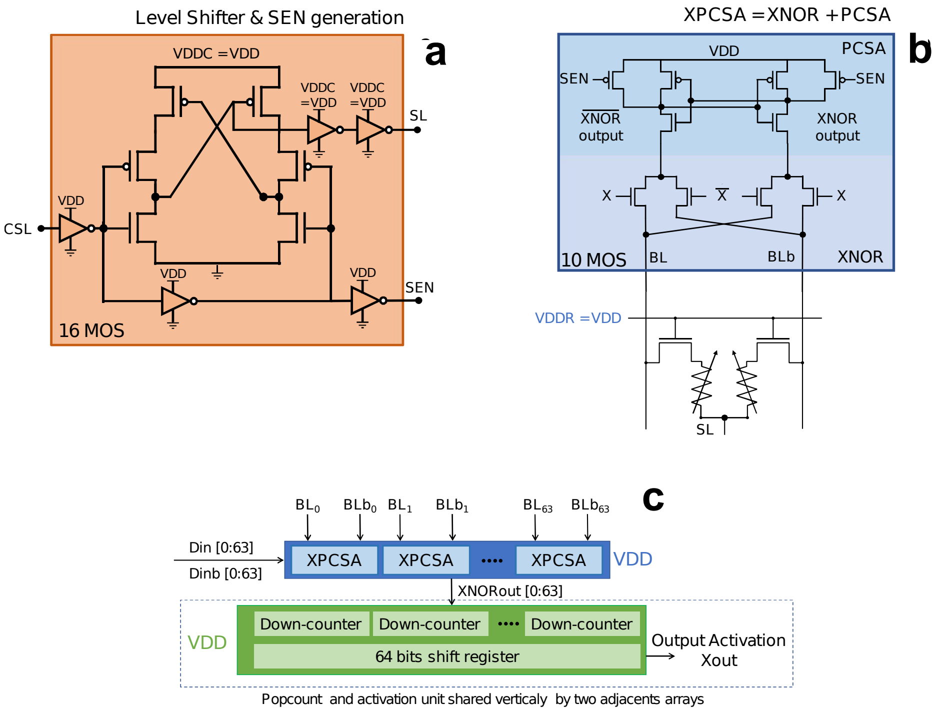

Supplementary Figure 3. Pipelined inference operation of the binarized neural network. a Detailed schematics of the Level shifter used to drive a given SL line in the array together with the sense enable (SEN) signal to drive the XPCSA read operation. During the idle and the inference states all voltages in the array are set to VDD (VDDC = VDD). **b** Detailed schematics of the Pre-charge sense amplifier associated to the XNOR layer to form the XPCSA circuit connected to BL and BLb array lines during the inference operation. During the sense operation the BL and BLb programming level shifter are disconnected and the selected WL (WL voltage is equal to VDDR=VDD) is polarized to VDD whereas the unselected WL are grounded. **c** Block scheme of the connection between the XPCSAs, that perform the XNOR between the weights of the selected WL and the activation input bit extended to 64 bits and applied to the XNOR layer through Din and Dinb vectors, and the digital block, with down-counters referred to as neuron register in the main article, that performs popcount and generate output neurons activation.

difference close to zero, depending on their resistance value. This property preserves the integrity of the value stored in the memristors, avoiding any read disturb effect during the charge phase.

- The finite state machine sets the control signal CSL to zero, which disconnects the BL and BLb from the PCSA and leaves them in a high impedance state. With CSL equals to zero, the SL is grounded, discharging both BL and BLb through the memristors and the XNOR layer with different time constants depending on the memristor resistance value. This difference in discharge time allows the latch in the PCSA to acquire the XNOR value. The small voltage difference between the electrodes of the memristors, together with short time constant, prevent memristors from facing any read disturb effect.

With each activated WL and activation input, and thus with each XNOR values, digital down-counters (referred to as neuron register in the main article) one per column or output neuron, perform the popcount operation by subtracting one from the threshold value when the XNOR value is equal to one. When all WLs have been activated, the popcount is finished, and by comparing the value of each down-counter to zero, it is straightforward to generate the output activation value of each neuron and to store them in an output shift-register (see Supplementary Figure 3b).

The activation process for the 128 output neurons driven by 116 inputs, in our engine, necessitates 1221 clock cycles. Each activation calculation itself takes five cycles and the operations involving reading through the XPCSA and generating the XNOR value for a specific line of memristors take 19 cycles. The resulting bandwidth, for each memory array, spans from 1.04 millions neuron activation computations per second at 10 MHz to 6.9 millions at 66 MHz. The performance could be further increased, e.g., by selecting WL and performing precharge all in one clock cycle. We avoided such optimization to prioritize the functionality of the test chip. Also, we made the choice, within our circuit, to synchronize all read phases with the system

clock, ensuring robust operation across various operating points. More conventional memory controllers, such as those based on integrated pulse generators[1] may allow larger throughput, but exhibit sensitivity to voltage and frequency variations.

Since all activations are coded with a single bit and stored in a shift register, shifting out, in a pipelined style, a single bit allows to provide activation value to the next neuron layer. Furthermore, with this pipelined process, all neuron layers are activated in parallel, with minimal data movement.

Supplementary note 4: Resource-efficient binarized neural network mapping strategy: Divide-and-conquer

This note describes the Divide-and-conquer strategy that we use to map neural networks to our chip, taking into account the resource constraints of the array. The challenge lies in accommodating for the fact that memristor arrays have a finite number of rows for input values, while a neural network may have a larger number of neurons in a layer. Our solution involves partitioning the inputs and performing the popcount operation on each partition individually, producing a partial output. These partial outputs are then combined to obtain the final output of that layer. The specific implementation techniques differ between fully connected and convolutional architectures, and we provide a detailed account of these strategies here.

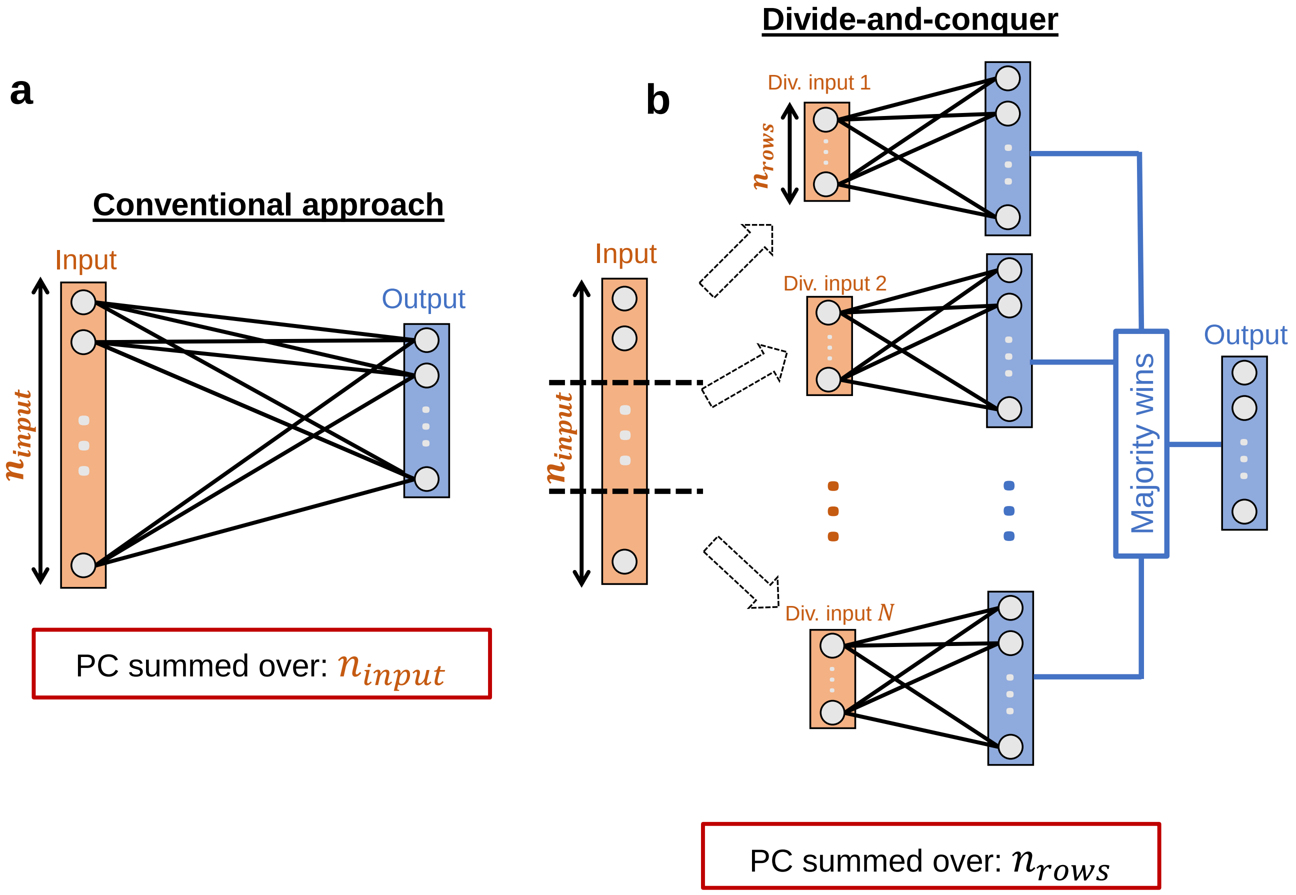

Supplementary Figure 4. Schematic for the Divide-and-conquer strategy for a fully connected architecture a A conventional fully connected layer of a neural network where the popcount operation is performed over n_{input} values of neurons. **b** Our Divide-and-conquer approach for the same neural network layer. The input layer is divided into N partitions with n_{rows} neurons in each, independently connected to output layers. These intermediate output layers are combined in a 'majority wins' function to produce the final output. Here, the popcount is over n_{rows} values.

Divide-and-conquer in fully connected architecture

The neural network operation of calculating the output from n_{input} number of inputs, without any circuit consideration, has the following form

$$X_{out,j} = \text{sign}\left(\sum_{i=1}^{n_{input}} XNOR\left(W_{ji}, X_{in,i}\right) - T_j\right). \tag{1}$$

In our case, the number of values over which we can perform the popcount operation is fixed by the number of rows present in our array (n_{rows}). Therefore, as shown in Supplementary Fig. 4, we divide the n_{input} into N partitions with n_{rows} units in each (hence, $N = \frac{n_{input}}{n_{rows}}$). If W_{ji}^r, $X_{in,i}^r$, and T_j^r represents the weights, inputs, and thresholds for the rth partition, the output is then calculated as

$$X_{out,j} = \text{sign}\left(\sum_{r=1}^{N} \text{sign}\left(\sum_{i=1}^{n_{rows}} XNOR\left(W_{ji}^r, X_{in,i}^r\right) - T_j^r\right)\right). \tag{2}$$

In Supplementary Fig. 4, the summation of the intermediate outputs and the following sign function is represented by the 'majority wins' function: if the +1s are in the majority among the outputs, it yields +1, and -1 otherwise. Since we are combining the intermediate outputs in this manner, N must be an odd number; otherwise, some outputs could have an equal number of +1s and -1s. We achieve this by choosing n_{inputs} such that N is odd.

The Divide-and-conquer strategy is used for the first hidden layer with 1,102 neurons as input in the fully connected architecture. For our array, among the 64 rows, a total of six rows are reserved to represent the threshold value, leaving a total of 58 rows available for the input values, which leads to N being equal to 19.

The Divide-and-conquer mapping strategy keeps the total number of weights in the network the same as a conventional implementation. Still, it involves a loss of information as it performs the summation in parts, and we suffer a slight degradation in accuracy (98.0% to 97.2% on MNIST).

Divide-and-conquer in convolutional architecture

For convolutional neural networks, the popcount operation is performed differently. In our approach, we use filters with dimensions of $3\times3\times N_C$ to convolve over the input feature map, where N_C represents the number of channels. To address the resource constraints of our array, we partition the input feature maps along the channel dimension and apply the appropriate number of corresponding filters, as shown in Fig. 5. Unlike in the case of fully connected neural networks, the number of channels in each partitioned feature map, denoted by N_{div}, is not equal to n_{rows}. Instead, owing to the 3×3 filter shape, N_{div} is given by the expression:

$$N_{div} = \left\lfloor \frac{n_{rows}}{9} \right\rfloor. \tag{3}$$

The total number of partitions is given by $N = \frac{N_C}{N_{div}}$, and the number of popcount operations performed in each block is $3 \times 3 \times N_{div}$. The intermediate outputs obtained from each division are combined in the same way as in the fully connected network to generate the output feature map. For the same reasons as the fully connected architecture, we choose the total number of filters N_C such that the total number of partitions N is odd.

For the convolutional (feature-extracting) part of our network, we used a value of N_{div} = 6 because of our n_{rows} size of 58 and eq. 3, and for the fully connected (classifier) part we used mapping as described in the previous section. The following table lists the number of partitions used for each of the layers:

Type of layer	N_C	N
Conv	198	33
Conv	354	59
Conv	738	123
FC	406×3×3	63
FC	1102	19

Also, the total number of filters, or in other words, learnable weights, are preserved as compared to an undivided architecture, although with some degradation of accuracy (90.0% to 86.6%).

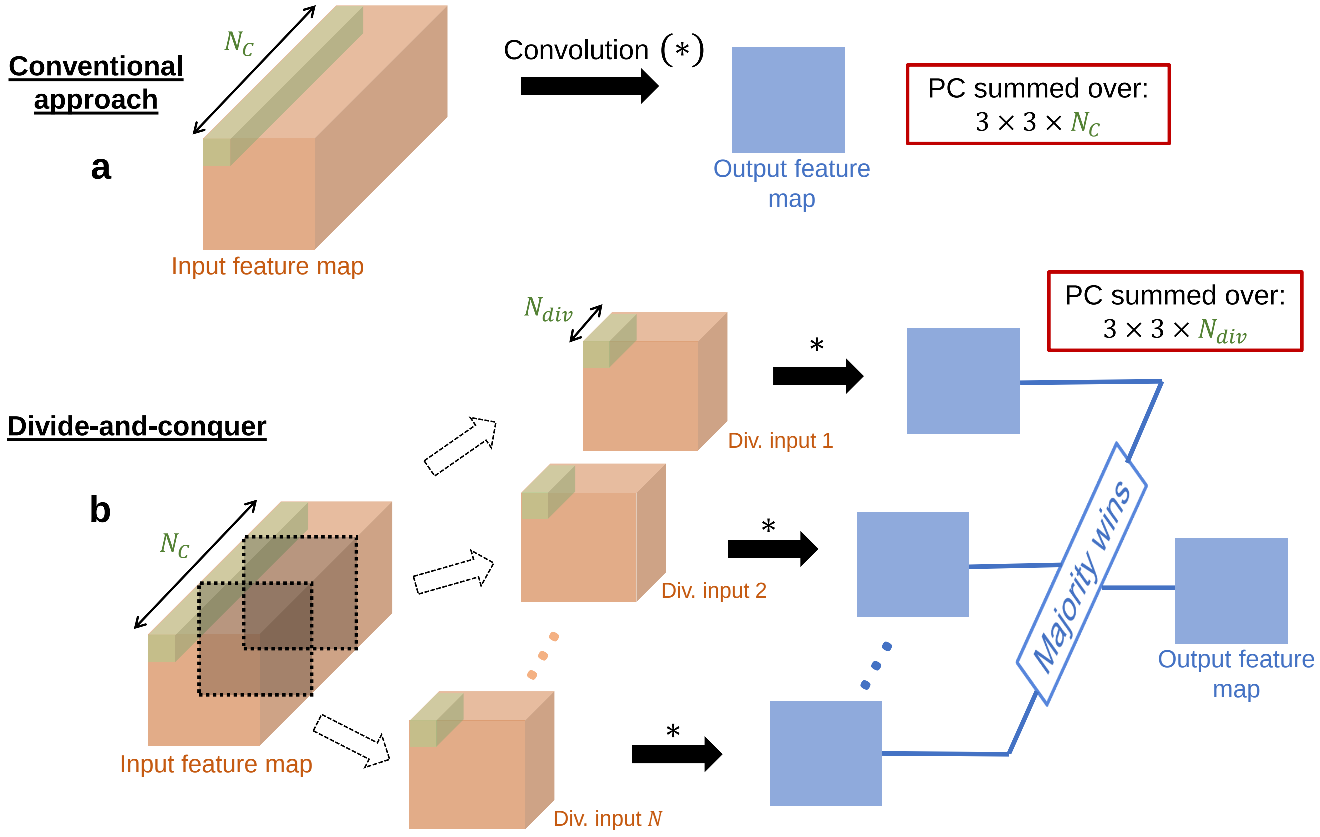

Supplementary Figure 5. Schematic for the Divide-and-conquer strategy for a convolutional architecture a In a conventional convolutional architecture, the input feature map with a channel-wise dimension of N_C is convolved with a filter of dimensions $3\times3\times N_C$ to produce the output feature map. Here, the popcount is done over $9N_C$ values. **b** In our Divide-and-conquer strategy, both the input feature maps and the filters are divided along the channel dimension into N partitions, each with N_{div} channels. Subsequently, the convolutions are performed on each of them, and the outputs are combined using a majority function to yield the final output. The popcount is performed over $9N_{div}$ values in this case.

Supplementary note 5: Comparison with state-of-the-art analog in-memory computing approaches

In this Note, we present a comparison between our binarized neural network (BNN) circuits and several state-of-the-art in-memory computing circuits based on memristor/RRAM[1,2], MRAM[3], and PCM[4], published since 2021. The main points of this comparison are summarized in Supplementary Table 1.

Each of these works employs an analog approach to perform accumulation computations. In refs.[1,2,4], a popular approach is adopted, where devices are connected in parallel, and accumulation is carried out using Kirchhoff's current law. Conversely, in[3], the devices are arranged in a series configuration, and accumulation is achieved through the summation of MRAM resistance values.

In-memory analog computation offers high efficiency; however, it necessitates a sophisticated CMOS periphery for reliable operation. Approaches in the literature to address this challenge vary significantly. Refs.[1,3,4] read multiple rows in parallel, enabling the simultaneous accumulation of numerous inputs. Nonetheless, the output remains analog, necessitating a conversion to digital. This conversion is achieved through area and energy-intensive CMOS analog-to-digital[1,2,4] or time-to-digital converters[3]. In ref.[2], only a partial sum is performed in-memory, circumventing the need for an analog-to-digital converter and opting for an efficient dedicated sense amplifier instead. Consequently, the full sum must be computed outside of the memory.

Refs.[2,3] employ devices as single-level cells (SLCs), significantly simplifying their programming and operation. This is the only feasible option in the case of[3], as MRAM cells can only exist in parallel or antiparallel states. In contrast, Refs.[1,4] utilize devices in an analog configuration, necessitating more sophisticated programming strategies. To enhance accuracy, Ref.[4] adopts a large-area, eight-transistor-four-memory-device (8T4R) basic cell.

Owing to the analog nature of computation, all these designs are tailored for a specific supply voltage and necessitate a

calibration step to compensate for device and circuit imperfections or variability[1–4]. This characteristic renders these circuits poorly suited for use with an unreliable power supply, such as a low-power energy harvester. Ref.[2] employs the most sophisticated circuits; this design uses the largest memory array, thus rendering it more susceptible to these issues.

All of these works report their energy efficiency in terms of tera-operations per second per watt (TOPS/W). However, comparing these figures presents a challenge due to various factors. The different implementations employ diverse precision levels for inputs, outputs, and weights, with some performing only partial accumulation. Additionally, the extent of functionality included on-chip varies, and the designs are fabricated on CMOS nodes ranging from 14 to 130 nanometers. Furthermore, not all periphery functionality is consistently incorporated in the reported TOPS/W. Despite these challenges, Ref.[3] reports the highest energy efficiency (up to 405 TOPS/W), which is expected, given its utilization of single-bit resolution for inputs and weights, and deployment of an advanced 28-nanometer CMOS node.

Our work diverges significantly from these implementations. Accumulation is performed entirely digitally, utilizing digital down-counters, while multiplication is executed within energy-efficient precharge sense amplifiers that read the memory states. Owing to this simplicity and the absence of analog computation, our system does not require calibration and can operate with various supply voltages (0.7 to 1.2 volts), functioning effectively even with a low-quality power supply. This resilience serves as its primary distinction from analog in-memory computing designs and is demonstrated in the main article by connecting our circuit to a miniature solar cell. We see that under extremely low illumination, the system's accuracy diminishes, yet it remains fully functional.

Our system is in principle less efficient than analog implementations, but this is to a large extent compensated by the absence of complex periphery circuitry. The measured energy efficiency is 2.9 TOPS/W, encompassing all elements of the system, except for the control finite state machines (refer to the Methods section in the main paper). To ensure functionality, we designed our system without clock gating, resulting in significant unnecessary energy consumption. We calculated the energy efficiency of a clock-gated system to be 22.5 TOPS/W (see Methods in the main paper), which is less efficient than the analog system[1] fabricated in a similar 130-nanometer technology node (capable of reaching up to 42 TOPS/W). Nevertheless, our design, unlike analog approaches, exhibits excellent scalability of its energy consumption. Utilizing the physical design kit of a fully-depleted silicon-on-insulator 28-nanometer CMOS process, we determined that the energy efficiency of a clock-gated design would achieve 397 TOPS/W (see Methods in the main paper). This performance is on par with the MRAM-based analog circuit of ref.[3], which is fabricated in a 28-nm technology node and also employs one-bit precision for inputs and synaptic weights. Note that our design and the one of ref.[3] are at an advantage with regard to the other designs of the table: as they only support binary activations, they do not need digital-to-analog converters. Analog implementations of binary-activations neural networks could also, in principle, avoid analog-to-digital converter. It could indeed be replaced by a comparison between two lines, for example in a binary network implementation using a differential approach for weights and activations on two complementary bitlines. This last approach might still have limitations due to the current density in both branches for high number of inputs. Circuits with analog-to-digital converter can deal with high number of inputs by calculating partial sums sequentially. These results suggest that our approach, in addition to its robustness, also offers high energy efficiency in scaled CMOS nodes.

	**This work**	Wan et al., Nature 2022[1]	Xue et al. Nat. Electron. 2021[2]	Jung et al., Nature 2022[3]	Khaddam et al., IEEE JSSC 2022[4]
Device	**HfOx/Ti Memristor**	HfOx/TaOx Memristor	Proprietary RRAM	MRAM	PCM
CMOS node	**130 nm**	130 nm	22 nm	28 nm	14 nm
Unit cell	**2T2R**	1T1R	1T1R	2T2R	8T4R
Levels per cell	**SLC**	Analog	SLC	SLC	Analog
Weight bit width	**1**	Analog	1-4	1	Analog
Input bit width	**1**	1-8	2-4	1	8
Preactivation bit width	**6**	3-10	6-11	4	8
Read circuit	**XPCSA**	Analog + ADC	Sense amplifier	Analog + TDC	Analog + ADC
Number of unit cells	**4× 64×64**	$48 \times 256 \times 256$	$1,024 \times 2,048$	64×64	256×256
Multiplication	**Digital**	Analog	Analog	Digital	Analog
Accumulation	**Digital**	Analog	Analog	Analog	Analog
Inference voltage	**Flexible (0.7-1.2 V)**	Predet.	Predet.	Predet.	Predet.
Need for calibration	**No**	Yes	Yes	Yes	Yes
Reported energy efficiency (TOPS/W)	**2.9 (measured)**<br>**22.5 (clock-gated)**<br>**397 (28-nm projection)**	7 to 43	37 to 146	262 to 405	10.5

Supplementary Table 1. Comparison of the design choices of our binarized neural network with leading emerging memory-based realizations of neural network hardware blocks. Abbreviations. PCM: Phase Change Memory. ADC: analog-to-digital converter. SLC: single-level cell. TDC: time-to-digital converter. Predet.: Predetermined. The content of this Table is discussed extensively within Supplementary Note 5.

Supplementary note 6: Analysis of the error sources

The main paper shows that when powered with a low supply voltage or a lowly-illuminated solar cell, our system can show bit errors. In this note, we present a detailed analysis of the sources of error in our chip, whether in terms of erroneous reading of weights during the XNOR operation or propagation faults in the digital part, with non-compliance with hold times and set-up times.

To that purpose, we first investigate the distribution of the resistance of programmed memristors. Our chip does not allow direct access to the memory cells. Therefore, to extract the distributions of the resistance states, we used a test chip manufactured using the same process and with the same 2T2R organization. For these new tests, the programming conditions used were extracted from simulations corresponding to the level-shifter outputs in our main chip:

- Forming: 4.49 V
- Reset: 2.69 V
- Set: 2.7 V.

Programming time is ten microseconds for all three operations. For reading, we used a voltage of 85 mV, based on simulations of the read circuit (PCSA) of the main chip.

Measurements from 1,024 1T1R cells show the distributions of the low resistance state (LRS, blue) and the high resistance state (HRS, red) in Supplementary Fig. 6a. The HRS distribution has a mean value of 220 kΩ with a standard deviation of 145 kΩ, a minimal value at three sigmas of 18.9 kΩ and a maximal value at three sigmas of 1.2 MΩ. The LRS distribution has a mean value of 7.8 kΩ with a standard deviation of 678 Ω, a minimal value at three sigmas of 6.1 kΩ and a maximal value at three sigmas of 14 kΩ.

The ratio between the mean value of the HRS distribution and the mean value of the LRS distribution is equal to 28.2, whereas the minimum ratio at three sigmas is equal to 1.35. These ratios are specific to the conditions in our chip; they could be adjusted by modifying programming conditions, either in terms of voltage amplitude or programming duration.

On this basis, we then simulated a memory array column together with the XNOR-augmented precharge sense amplifiers (XPCSA) circuit to analyze the sources of error at the weight level during inference, by varying the supply voltage and operating frequency. These SPICE-type simulations are based on Monte Carlo draws, including the transistors' global and local variability and memristors' measured variability (1000 draws per supply and frequency condition) for each operating point.

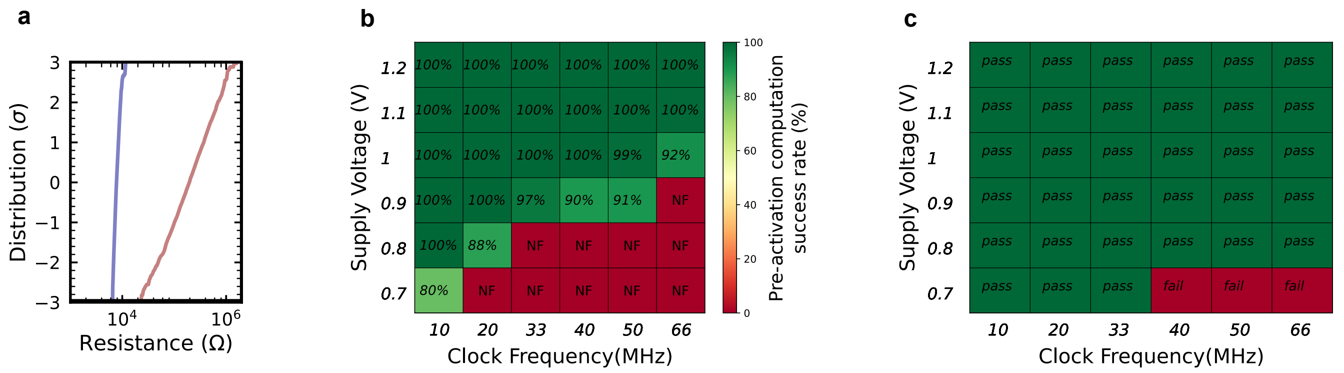

Supplementary Figure 6. **Investigations concerning error sources in our design.** **a** Cumulative Distribution Function of the two resistance states: low (LRS, blue) and high (HRS, red), measured on 1,024 memristors with programming conditions mimicking the ones used in the binarized neural network integrated circuit. **b** Simulated shmoo plot, presenting preactivation success rate (XPCSA output), for different operation frequencies and supply voltages. They were obtained by simulating a memory array column together with the XNOR-augmented precharge sense amplifiers (XPCSA) circuit. For each operating point, 1000 Monte-Carlo draws are simulated, taking into account local and global variations of MOS transistors and memristor variability extracted from the LRS and HRS distribution measured on 1024 memristors and reported in Suppl. Fig. 6a. NF means non-functional. **c** Simulated shmoo plot, presenting popcount register successful updates, for different operation frequencies and supply voltages. They were obtained by simulating electrically the popcount register of our integrated circuit, post place-and-route, in all process corner case scenarios. Pass means successful update, Fail means unsuccessful update.

In Supplementary Fig. 6b, we present a shmoo plot based on these simulations, showing the proportion of successful preactivation computation value (output of the XPCSA) for various operation frequencies and supply voltages, for a balanced pattern in terms of weights and inputs activation values (Din, Dinb). As expected, the preactivation computation is degraded for high frequency and low supply voltage. The interpretation of the simulations shows that when decreasing the supply voltage and/or increasing the frequency, the errors first tend to appear depending on the ratio between the HRS and LRS resistances, which fluctuates due to memristor variability. Specifically, when the ratio between HRS and LRS values is low due to a low HRS resistance, the PCSA charge/discharge time is only mildly affected. However, with a low ratio due to a high LRS value, the charge and discharge times become significantly impacted, which can again result in reading errors: the voltages of the two branches of the sense amplifier do not differentiate well. In this situation, the mismatch in the properties of the memristor selection transistor and of transistors of the sense amplifier can cause errors. This type of error is observed in the green non-100% cases in Supplementary Fig. 6b. When decreasing the supply voltage and/or increasing the frequency further (red cases in Supplementary Fig. 6b), the charge operation of the sense amplifier is not complete, and no read operation occurs, leading to a non-functional circuit.

The error rates are higher than those seen in the experimental shmoo plot (see main article, Fig. 3a), which is expected, as the Monte Carlo simulations include global and local variation in the MOS transistors, whereas the fabricated chip shows only local variations. The simulations, therefore, model a worst case, which we would observe on some outlier chips if we fabricated circuits on multiple wafers. Moreover, measurements are based on output activation success rate, thus the comparison between popcount and threshold partly balances the preactivation errors' influence on activation values.

To complete the error analysis of our solution, we carried out SPICE-type simulations of the popcount register, using a circuit including parasitics extracted from our chip after placement and routing. Registers are the most critical digital parts of our circuit. We simulated the update of the popcount register, for various supply voltages and operating frequencies, and for all the process corner cases (as the functionality of registers does not depend on transistor matching, Monte Carlo simulation is not needed in this case). Supplementary Fig. 6c shows the pass/fail results of this set of corner case simulations for the various conditions of supply voltage and frequency. The results show more robust behavior than in Supplementary Fig. 6b: fail situations only appear for the most reduced supply voltage (0.7 V) and above 33 MHz.

The analysis of both sets of simulations clearly shows the robustness of the preactivation computation based on our differential reading scheme with the XPCSA, and also the robustness of the digital part. We conclude that in most conditions, errors occur when reading the memristors due to the variability in the MOS process associated with distribution tail of memristor resistance values. For the lowest voltage (0.7 V) and for frequencies above 33 MHz, our BNN engine is also non-functional due to limitations introduced by the digital part of the circuit.

Supplementary note 7: Estimation of the error rate of an in-memory computing solution

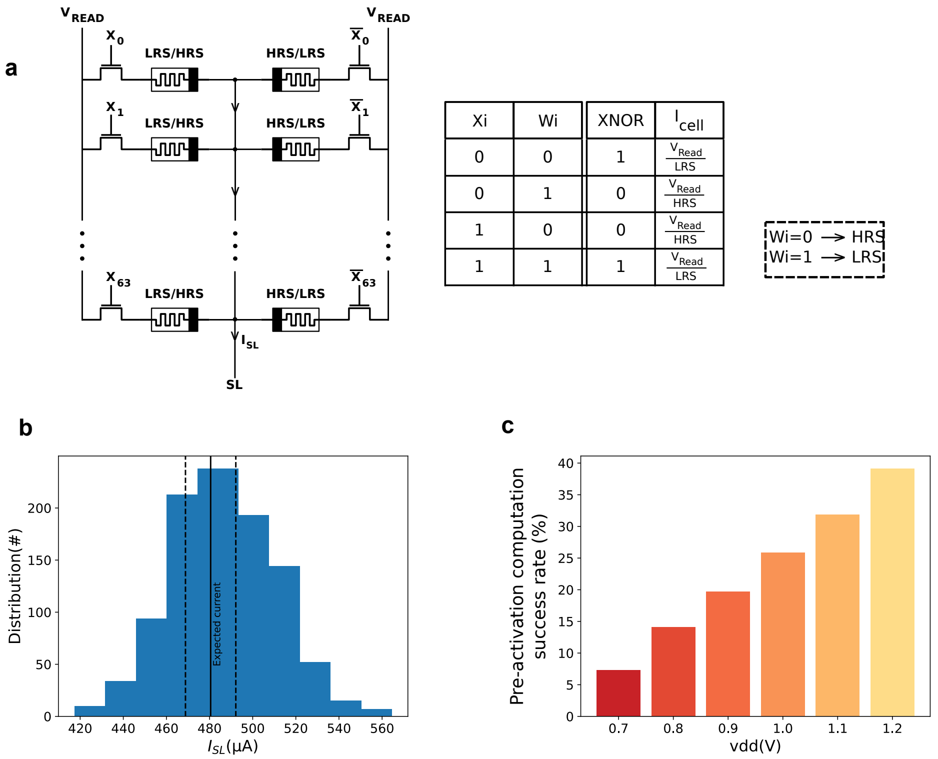

Supplementary Figure 7. Simulations of an in-memory computing solution with binary coding. a Schematic of the simulated in-memory computing solution based on current summation, using binary coding. The select transistors and the memristors are the same as the ones used in our solution. The read voltage V_{READ} is set to 0.5 V. **b** Simulated distribution of the current summation on the source line (SL). They were obtained by simulating a memory array column without any readout circuit, therefore neglecting the additional variability that such circuitry would cause. For each operating point, 1000 Monte-Carlo draws are simulated based on local and global variations for MOS transistors and memristors variability extracted from the LRS and HRS distribution measured on 1024 memristors, reported in Suppl. Fig. 6a. Supply voltage is 1.2 V. **c** Simulated histogram, presenting preactivation success rate for different supply voltage.

In this note, for comparison purposes, we introduce an evaluation of an equivalent analog in-memory computing solution based on Kirchhoff's laws, with binary coding of activations and weights. To make the comparison as fair as possible, we considered an analog in-memory computing solution with differential coding of the weights, as in our digital solution, initially proposed for use with MRAM in ref.[5]. +1 and -1 weights are coded with respectively HRS/LRS and LRS/HRS values. The input activation is also coded differentially and drives directly the select transistor gates, as represented schematically in Supplementary Fig. 7a. The preactivation value is obtained using the Kirchhoff current law, as the sum of each bit-cell current collected on the vertical source line (SL).

To evaluate this solution, we performed SPICE-type simulations with the same conditions as for the simulation of our solution in Suppl. Note 6. We simulate a full column of 64 2T2R memory cells. We did not include any reading solution. Read circuitry would necessarily cause additional variability. Our simulation, therefore, corresponds to a best-case scenario for analog in-memory computing. The simulation results showed no dependency versus the frequency, meaning that the activation time that we chose is sufficient to stabilize the current on the SL source line, regardless of the frequency.

Suppl. Fig. 7b compares the SL current distribution, obtained from the 1000 Monte Carlo draws for each operating point, to the ideal expected interval. This distribution was obtained using a nominal supply voltage vdd (1.2 V) and a computation with balanced XNOR results (32 ones and 32 zeros). Only 39% of the results fall within the expected range, or in other words, the preactivation is computed correctly only 39% of the cases.

Suppl. Fig. 7c presents the proportion of correctly-computed preactivation values, when varying the supply voltage. This Figure is plotted using the same conditions as Suppl. Fig. 6b to allow for a direct comparison with our solution. Decreasing the supply voltage strongly reduces the success rate: it reaches 7% for a supply voltage of 0.7 V. The impact of the supply voltage is due to the fact that MOSFET variation increases when the supply voltage approaches the transistor threshold voltage,

which is high (near 0.6 V) in the low-power CMOS process that we are using. These results are in sharp contrast with the ones obtained for our approach under the same conditions (Suppl. Fig. 6b), where 100% accuracy on preactivation values can be obtained for supply voltages down to 0.8 V, and 80% accuracy for a supply voltage of 0.7 V. This comparison demonstrates the high robustness of our method with regard to analog in-memory computing, even if using binary coding of the weights. Note that the leading source of errors in Suppl. Fig. 7b is the process variation of transistors. In a practical setting, this type of variation can be partly compensated by using circuit calibration, which needs to be supply voltage-dependent. Most real-life analog IMC circuits, therefore, use calibration[1,2,5,6]. Our circuit, by contrast, functions without any calibration, for a wide range of supply voltages and process variation.

Supplementary note 8: Alternative designs to use energy harvesters as stable power sources

In the main manuscript, we demonstrate that our circuit, due to its exceptional robustness, can be directly powered by a solar cell. This is the case even under minimal illumination, albeit with a slight compromise on the programmed AI's accuracy. One potential alternative would be to integrate a full power management unit (PMU) between the energy harvester and the AI circuit. This approach would permit a less robust design style for the AI circuit, such as analog in-memory computing. This note describes usable state-of-the-art power management solutions that yield a consistent supply voltage from a harvesting source, offering a basis for comparison with our approach.

Supplementary Table 2 presents a comprehensive overview of the salient features of PMUs in representative published solar-cell-harvested systems. For a stable VDD power supply when powered by a solar cell, key components of a PMU would include a DC-DC converter, a Maximum Power Point Tracking (MPPT) algorithm, and an auxiliary energy storage unit[7]. There are various design paths to consider. The choice between switched-capacitor-based or inductance-based voltage converter architectures can be based on the system requirements and solar cell output potential[8]. Storage options range from batteries, offering extended storage, to capacitors suitable for sporadic operations. The PMU strategy can be dynamic, either powering the chip while simultaneously charging the battery or leveraging the battery as a backup power source.

Incorporating a PMU is not without challenges. Predominantly, it would lead to an enlarged chip area and increased system complexity. Considerations would include storage capacities, switches, and the tangible realization of the MPPT algorithm. At the system level, provisions for external inductors and capacitors – if they are not integrated – and potentially batteries, are indispensable for achieving outputs in the tens of milliwatts domain[7,9]. Wholly integrated solutions, as described in ref.[10,11], are restricted to outputs ranging from tens to hundreds of microwatts, but come at a considerable area expense. Still, the PMU proposed by ref.[11], could provide to our chip a stable supply voltage of one volt for a maximal frequency of 10 MegaHertz, based on its available output power. Another concern across these solutions is energy dissipation, as highlighted in Supplementary Table 2, wasting of part of the harvested energy. Our approach stands out for its ability to circumvent these issues, which underlines its uniqueness.

	Chen et al., IEEE ISSCC 2015[7]	Lu et al., IEEE ISSCC 2016[9]	Rawy et al., IEEE JSSCC 2018[11]	Liu et al., IEEE JSSCC 2015[10]
CMOS node	500 nm	350 nm	65 nm	180 nm
Fully integrated	No	No	Yes	Yes
External Inductor	4.7μH	22μH	NA	NA
External Capacitor	4.7μF	4.7μF	NA	NA
MPPT mechanism	PFM	PFM	SFM and CRM	SFM, CRM and SWM
Die area	0.5 mm^2	4 mm^2	0.5 mm^2	1.69 mm^2
Input Voltage	3.6V to 4V	0.03V to 3.6V	0.35V to 1V	0.5V to 1.6V
Output Voltage	1V to 3.3V	3.6V	1V	1.8V
Output Power	1μW - 15mW	-	100nW to 300μW	< 35μW
Peak efficiency	93%	85%	88%	72%

Supplementary Table 2. Comparison of state-of-the-art power management units, with or without external components, operating with a single input solar cell to power a single load. The content of this Table is discussed within Supplementary Note 8.

Supplementary References

1. Wan, W. *et al.* A compute-in-memory chip based on resistive random-access memory. *Nature* **608**, 504–512 (2022).
2. Xue, C.-X. *et al.* A cmos-integrated compute-in-memory macro based on resistive random-access memory for ai edge devices. *Nat. Electron.* **4**, 81–90 (2021).
3. Jung, S. *et al.* A crossbar array of magnetoresistive memory devices for in-memory computing. *Nature* **601**, 211–216 (2022).
4. Khaddam-Aljameh, R. *et al.* Hermes-core—a 1.59-tops/mm 2 pcm on 14-nm cmos in-memory compute core using 300-ps/lsb linearized cco-based adcs. *IEEE J. Solid-State Circuits* **57**, 1027–1038 (2022).
5. Deaville, P., Zhang, B., Chen, L.-Y. & Verma, N. A maximally row-parallel mram in-memory-computing macro addressing readout circuit sensitivity and area. In *ESSCIRC 2021-IEEE 47th European Solid State Circuits Conference (ESSCIRC)*, 75–78 (IEEE, 2021).
6. Hung, J.-M. *et al.* A four-megabit compute-in-memory macro with eight-bit precision based on cmos and resistive random-access memory for ai edge devices. *Nat. Electron.* **4**, 921–930 (2021).
7. Chen, H. J., Wang, Y. H., Huang, P. C. & Kuo, T. H. An energy-recycling three-switch single-inductor dual-input buck/boost dc-dc converter with 93% peak conversion efficiency and 0.5 mm2 active area for light energy harvesting. In *2015 62nd IEEE International Solid-State Circuits Conference, ISSCC 2015-Digest of Technical Papers*, 374–375 (Institute of Electrical and Electronics Engineers Inc., 2015).
8. Newell, D. & Duffy, M. Review of power conversion and energy management for low-power, low-voltage energy harvesting powered wireless sensors. vol. 34, 9794–9805 (IEEE, 2019).
9. Lu, Y., Yao, S., Shao, B. & Brokaw, P. 21.3 a 200na single-inductor dual-input-triple-output (dito) converter with two-stage charging and process-limit cold-start voltage for photovoltaic and thermoelectric energy harvesting. In *2016 IEEE International Solid-State Circuits Conference (ISSCC)*, 368–369, DOI: 10.1109/ISSCC.2016.7418060 (2016).
10. Liu, X. & Sánchez-Sinencio, E. An 86self-sustaining pv energy harvesting system with hysteresis regulation and time-domain mppt for iot smart nodes. *IEEE J. Solid-State Circuits* **50**, 1424–1437, DOI: 10.1109/JSSC.2015.2418712 (2015).
11. Rawy, K., Yoo, T. & Kim, T. T.-H. An 88w energy harvesting system with 3-d mppt using switch width modulation for iot smart nodes. *IEEE J. Solid-State Circuits* **53**, 2751–2762, DOI: 10.1109/JSSC.2018.2833278 (2018).
